# Multiple Chronic Conditions Among US Adults: A 2012 Update

**DOI:** 10.5888/pcd11.130389

**Published:** 2014-04-17

**Authors:** Brian W. Ward, Jeannine S. Schiller, Richard A. Goodman

**Affiliations:** Author Affiliations: Jeannine S. Schiller, Centers for Disease Control and Prevention, Hyattsville, Maryland. Richard A. Goodman, Centers for Disease Control and Prevention, and the Emory University Division of General Medicine and Geriatrics, Atlanta, Georgia.

## Abstract

The objective of this research was to update earlier estimates of prevalence rates of single chronic conditions and multiple (>2) chronic conditions (MCC) among the noninstitutionalized, civilian US adult population. Data from the 2012 National Health Interview Survey (NHIS) were used to generate estimates of MCC for US adults and by select demographic characteristics. Approximately half (117 million) of US adults have at least one of the 10 chronic conditions examined (ie, hypertension, coronary heart disease, stroke, diabetes, cancer, arthritis, hepatitis, weak or failing kidneys, current asthma, or chronic obstructive pulmonary disease [COPD]). Furthermore, 1 in 4 adults has MCC.

## Objective

From 2001 through 2010, the prevalence of persons with multiple (≥2) chronic conditions (MCC) in the United States increased; approximately 26% of US adults had MCC in 2010, when 10 different conditions (ie, hypertension, coronary heart disease, stroke, diabetes, cancer, arthritis, hepatitis, weak or failing kidneys, asthma, and COPD) were considered (1). The large and growing prevalence of MCC has prompted a spectrum of responses — including a national initiative calling for better research and data on MCC — to address this clinical and public health problem (2–5). In response to the need for ongoing surveillance of chronic conditions and to more frequently monitor their prevalence, we analyzed data from the 2012 National Health Interview Survey (NHIS) to produce updated estimates of single chronic conditions and MCC among the noninstitutionalized, civilian US adult population.

## Methods

The NHIS is a multistage health survey of the US civilian, noninstitutionalized population (6,7). Information from the NHIS Family Core questionnaire (Family Core) on sex, race/ethnicity, age, and health insurance coverage was used in this analysis. In the Family Core, 1 adult from the family self-reports and acts as a proxy for other family members. Data on chronic conditions are collected by using the Sample Adult Core questionnaire. The respondent (ie, “sample adult”) is randomly selected from all adults in the family aged 18 years or older. A proxy is used for the sample adult only if a health condition makes it impossible for the sample adult to respond for himself or herself (6,7). Our analyses include 34,525 sample adults from the 2012 NHIS (final response rate: 61.2%).

Adults were identified as having 0, 1, 2, or 3 or more chronic conditions. The chronic conditions included in this study were 10 physical conditions from a list of 20 conditions identified by the US Department of Health and Human Services (HHS) to foster a more consistent and standardized approach to measuring the occurrence of chronic conditions in the United States (3). Participants were identified as having 1 of the 10 conditions if they had ever been told by a doctor or health care provider that they had hypertension, coronary heart disease, stroke, diabetes, cancer, arthritis, or hepatitis; had experienced weak or failing kidneys during the past 12 months; currently had asthma; or had COPD. COPD was assessed by using responses from 2 survey questions asking adults if they had ever had emphysema or had had chronic bronchitis in the past 12 months; adults answering yes to either question were identified as having COPD. SUDAAN software 10.0.1 (RTI International, Research Triangle Park, North Carolina) was used to account for the complex sample design of the NHIS when generating estimates and confidence intervals. Estimates were calculated for subgroups defined by age, sex, race/ethnicity, and health insurance coverage to show prevalence of single conditions and MCC among these subgroups. Two-tailed significance tests were used to test for differences in prevalence between population subgroups; all differences reported are significant (*P* < .05).

## Results

In 2012, among civilian, noninstitutionalized US adults, approximately half (49.8%, 117 million) had at least 1 of 10 selected chronic conditions. More specifically, 24.3% had 1 chronic condition, 13.8% had 2 conditions, and 11.7% had 3 or more conditions (Table). Among adults with at least 1 chronic condition, more than half (approximately 60 million) had MCC.

**Table:** Estimated Percentage and Number of US Adults Aged ≥18 Years with Chronic Conditions^a^ by Select Characteristics, National Health Interview Survey, 2012

Characteristic	0 Chronic Conditions		1 Chronic Condition		2 Chronic Conditions		≥3 Chronic Conditions	
	% (95% CI)	Population^b^	% (95% CI)	Population^b^	% (95% CI)	Population^b^	% (95% CI)	Population^b^
Total^c^	50.2 (49.5–51.0)	118,000	24.3 (23.7–24.9)	57,154	13.8 (13.3–14.2)	32,350	11.7 (11.2–12.1)	27,416
**Sex**								
Male	52.2 (51.2–53.4)	59,078	24.1 (23.2–25.0)	27,248	13.0 (12.4–13.7)	14,685	10.7 (10.1–11.3)	12,060
Female	48.4 (47.4–49.3)	58,922	24.5 (23.8–25.3)	29,906	14.5 (13.9–15.1)	17,666	12.6 (12.0–13.2)	15,356
**Race/ethnicity**								
Non-Hispanic white	46.5 (45.5–47.5)	72,617	25.6 (24.9–26.4)	39,999	15.2 (14.6–15.8)	23,671	12.7 (12.2–13.3)	19,887
Non-Hispanic black	46.3 (44.5–48.2)	12,494	25.5 (24.0–27.1)	6,873	14.5 (13.3–15.7)	3,907	13.7 (12.6–14.9)	3,687
Non-Hispanic Asian	64.4 (61.4–67.3)	7,806	19.9 (17.7–22.2)	2,405	9.1 (7.6–10.9)	1,105	6.6 (5.4–8.1)	802
Non-Hispanic other race^d^	49.7 (45.0–54.5)	2,349	22.4 (18.6–26.7)	1,058	12.6 (9.8–16.0)	594	15.3 (12.4–18.7)	721
Hispanic	65.1 (63.5–66.6)	22,735	19.5 (18.2–20.9)	6,819	8.8 (8.0–9.7)	3,073	6.6 (5.9–7.5)	2,319
**Age group**								
18–44 y	73.5 (72.5–74.5)	81,620	19.4 (18.6–20.3)	21,590	5.1 (4.6–5.5)	5,616	2.0 (1.7–2.3)	2,208
45–64 y	37.1 (35.9–38.3)	30,440	30.6 (29.5–31.7)	25,100	18.5 (17.6–19.4)	15,168	13.8 (13.0–14.7)	11,329
≥65 y	14.2 (13.1–15.3)	5,940	25.0 (23.8–26.3)	10,464	27.6 (26.3–29.0)	11,566	33.2 (31.7–34.6)	13,879
**Health insurance coverage^e^**								
Private	52.2 (51.2–53.2)	76,808	25.3 (24.5–26.1)	37,216	13.1 (12.5–13.7)	19,298	9.4 (8.9–9.9)	13,844
Public	27.6 (26.2–29.0)	11,141	24.2 (22.9–25.6)	9,778	21.4 (20.2–22.6)	8,635	26.9 (25.6–28.2)	10,850
Other	49.1 (45.3–53.0)	3,702	26.8 (23.7–30.2)	2,021	13.2 (11.0–15.9)	997	10.8 (8.8–13.2)	814
Uninsured	65.9 (64.4–67.5)	26,260	20.4 (19.1–21.8)	8,126	8.7 (7.9–9.7)	3,473	4.9 (4.3–5.7)	1,959

Abbreviation: CI, confidence interval.

a Chronic conditions include hypertension, coronary heart disease, stroke, diabetes, cancer, arthritis, hepatitis, weak or failing kidneys, current asthma, and COPD.

b Population in 1,000s.

c Total 2012 US adult population: 234 million persons.

d Adults identifying as multiple races were included in the “other race” category.

e Health insurance coverage was based on a hierarchy of mutually exclusive categories (6,7). Public health insurance coverage includes Medicaid, Children’s Health Insurance Program, and Medicare. Other health insurance coverage includes state-sponsored health plans, other government programs, and military health plans.

The estimated prevalence of MCC varied by specific subpopulations of US adults. Women were more likely than men to have exactly 2 conditions or 3 or more conditions. The prevalence of MCC was higher among non-Hispanic white adults, non-Hispanic black adults, and non-Hispanic adults of other races than among non-Hispanic Asian adults and Hispanic adults. The percentage of adults with MCC (both 2 and ≥3) increased with age. Examination by health insurance coverage status indicated that the percentage of adults with 1 or more chronic conditions was lower among the uninsured than among adults with any type of coverage. MCC prevalence was also greater among adults with public coverage than among adults with private or some other type of coverage.

## Discussion

We used the 2012 NHIS to update earlier estimates of the prevalence of US adults with single chronic conditions and MCC. Results showed no significant decrease in the prevalence of adults who had MCC between the years 2010 (26.0%) (1) and 2012 (25.5%), a meaningful finding because previous analyses showed an overall significant, increasing trend in MCC prevalence before 2010 (1,8).

The NHIS estimates of the percentage of adults with one or more of the chronic conditions identified in the HHS standardized definition (3) are likely conservative; the NHIS captures only 10 of the 20 chronic conditions included in the list of chronic conditions standardized to promote more consistent measurement (3), and this list of 20 conditions is itself a subset of the entire universe of chronic conditions. Mental health conditions included in the list of 20 conditions were not included in the NHIS Core questionnaires and therefore were not included in our study. This is especially problematic when examining younger adults (9). Finally, it should also be noted that the NHIS samples are from the noninstitutionalized civilian population and therefore do not include persons in long-term care or other congregant settings among whom the prevalence of MCC may be higher.

These 2012 estimates show that 25.5% of US adults have MCC and that MCC remain a public health concern. Some of the primary goals of the national initiative focused on addressing MCC in the United States include strengthening health care and public health systems, improving self-care management of MCC, and providing better tools and information to health care providers (2,4). To help meet these goals, research is needed to inform gaps in knowledge on MCC (2,4), including an effort to more frequently monitor MCC across the US population by using data from national health surveys such as the NHIS and other surveillance systems (3). Such research can provide prevalence estimates to individuals leading prevention efforts and could improve the targeting of interventions.
